# Early postoperative decrease of skeletal muscle mass predicts recurrence and poor survival after surgical resection for perihilar cholangiocarcinoma

**DOI:** 10.1186/s12885-022-10453-2

**Published:** 2022-12-28

**Authors:** Sho Yasuta, Motokazu Sugimoto, Masashi Kudo, Shin Kobayashi, Shinichiro Takahashi, Masaru Konishi, Naoto Gotohda

**Affiliations:** Department of Hepatobiliary and Pancreatic Surgery, National Cancer Center Hospital East, 6-5-1, Kashiwanoha, Kashiwa, Chiba, 277-8577 Japan

**Keywords:** Sarcopenia, Perihilar cholangiocarcinoma, Major hepatectomy, Skeletal muscle mass volume, Survival outcomes

## Abstract

**Background:**

Preoperative sarcopenia is a predictor of poor survival in cancer patients. We hypothesized that sarcopenia could progress as occult metastasis arose, especially after highly invasive surgery for highly aggressive malignancy. This study aimed to evaluate the associations of postoperative changes in skeletal muscle mass volume with survival outcomes in patients who underwent surgery for perihilar cholangiocarcinoma.

**Methods:**

Fifty-six patients who underwent major hepatectomy with extrahepatic bile duct resection for perihilar cholangiocarcinoma were studied. The skeletal muscle index (SMI) at the third lumbar vertebra was calculated from axial computed tomography images taken preoperatively and 3–6 months postoperatively (early postoperative period). The associations of clinicopathological variables, including changes of SMI after surgery, with overall survival and recurrence-free survival were evaluated. Moreover, the associations of decreased SMI and elevated serum carbohydrate antigen 19–9 level with early recurrence and poor survival was compared.

**Results:**

Among 56 patients, 26 (46%) had sarcopenia preoperatively and SMI decreased in 29 (52%) in the early postoperative period. During the median follow-up of 57.9 months, 35 patients (63%) developed recurrence and 29 (50%) died. Decreased SMI in the early postoperative period was independently associated with a shorter overall survival (hazard ratio, 2.39; 95% confidence interval, 1.00–6.18; *P* = 0.049) and a shorter recurrence-free survival (hazard ratio, 2.14; 95% confidence interval, 1.04–4.57; *P* = 0.039), whereas elevated carbohydrate antigen 19–9 level was not.

**Conclusions:**

Decreased SMI in the early postoperative period may be used as a predictor for recurrence and poor survival in patients undergoing surgery for perihilar cholangiocarcinoma.

## Background

Perihilar cholangiocarcinoma is an aggressive malignancy arising from the biliary confluence, with the reported median overall survival of 4–6 months [1, 2]. While surgical resection is the only curative treatment for perihilar cholangiocarcinoma, median survival ranges from 19 to 39 months after surgery [3–6]. Major hepatectomy with extrahepatic bile duct resection and reconstruction (EBDR) is a standard but highly invasive surgery to remove perihilar cholangiocarcinoma, with a 40–70% postoperative morbidity rate and 5–15% mortality rate [7–11]. Accordingly, general weakness, malnutrition, and/or decreased quality of life are anticipated in patients especially early after this surgery.

Sarcopenia was defined as a syndrome characterized by progressive and generalized loss of skeletal muscle mass volume (SMV) and strength [12]. SMV is presumed to decrease in patients with aggressive malignancy, especially after highly invasive surgery, because of hypercatabolism, systemic inflammation, and insufficient protein synthesis [13, 14]. Such patients have risks of tumor progression, sarcopenia, and cachexia both pre- and postoperatively [13]. Most previous studies investigated the impact of preoperative sarcopenia on postoperative outcomes, whereas few have investigated the impact of postoperative sarcopenia on postoperative outcomes. We hypothesized that the postoperative change in SMV might more accurately reflect postoperative tumor progression than preoperative variables or pathological findings. Therefore, this study aimed to evaluate the associations of postoperative changes in SMV with survival outcomes in patients who underwent major hepatectomy with EBDR for perihilar cholangiocarcinoma and to determine whether decreased SMV was associated with poor survival.

## Methods

### Patients and clinicopathological data collection

The medical records of 78 consecutive patients who underwent major hepatectomy with EBDR for perihilar cholangiocarcinoma between May 2006 and June 2017 at our institution were reviewed. Perihilar cholangiocarcinoma was defined as a tumor located in the extrahepatic biliary tree proximal to the origin of the cystic duct, consistent with the Union for International Cancer Control (UICC) Classification 8th Edition [15]. In all patients, perihilar cholangiocarcinoma was diagnosed by radiological imaging and pathological confirmation of surgical specimens. The following patients were excluded from analysis: 2 patients who died secondary to postoperative complications, 10 patients lost to follow-up within 12 months postoperatively, and 11 patients who received adjuvant chemotherapy as part of a clinical trial (there was 1 patient who both lost to follow-up and received adjuvant chemotherapy). Therefore, 56 patients were investigated.

**Fig. 1:**
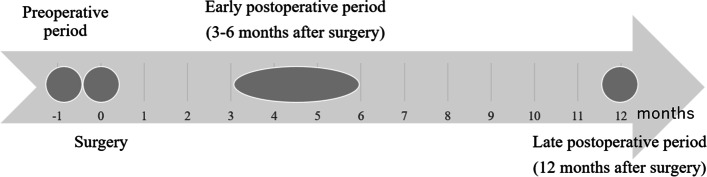
A timeline in this study. Clinicopathological data of 3 points, including preoperative period, early postoperative period (3–6 months after surgery), and late postoperative period (12 months after surgery)

Clinicopathological data, which could be affected survival or change of skeletal muscle mass, were retrieved from medical records. Those data included age, sex, body mass index (BMI), American Society of Anesthesiologists classification score, Charlson comorbidity index, preoperative biliary drainage, and portal vein embolization. Preoperative laboratory data included serum concentrations of total bilirubin, albumin, and carbohydrate antigen 19–9 (CA 19–9). Intraoperative factors included type of surgical procedure, operation time, blood loss, and blood transfusion. Postoperative complications included major complications consistent with Clavien-Dindo grade ≥ IIIa [16], surgical site infection, bile leakage, liver failure, and length of hospitalization. Pathological findings included tumor size, T factor, lymph node metastasis, and resection curability. Serum CA 19–9 level was measured 3–6 months (early postoperative period) and 12 months (late postoperative period) postoperatively (Fig. 1). Elevated serum CA 19–9 level was defined as > 37 U/mL.

This study was approved by the ethics committee of the National Cancer Center (#2018–053).

### Preoperative management, surgical technique, and postoperative follow-up

Computed tomography (CT), ultrasonography, magnetic resonance imaging, and endoscopic retrograde cholangiopancreatography were routinely performed for the diagnosis of perihilar cholangiocarcinoma. Preoperative biliary drainage was performed in patients with obstructive jaundice. When the serum total bilirubin concentration decreased to < 2 mg/dL after biliary drainage, liver function was evaluated by testing indocyanine green retention at 15 min. Indocyanine green retention at 15 min and CT volumetry were routinely examined to evaluate the functional reserve of the future liver remnant. Preoperative portal vein embolization was performed in patients who were judged to have insufficient future liver remnant volume relative to liver function [17, 18].

All patients underwent major hepatectomy with EBDR. In particular, regional lymph node dissection of the common hepatic artery and hepatoduodenal ligament were performed. Common bile duct was divided at the level of the pancreas and submitted for frozen section analysis to evaluate tumor negativity of the cut-end. Hemilateral hepatic artery and portal vein were divided. Hepatic parenchymal dissection was performed and caudate lobectomy was routinely performed. The proximal hemilateral bile duct was divided and a specimen was extracted. After confirmation of the tumor negativity of the proximal bile duct cut-end, hepaticojejunostomy with Roux-en-Y anastomosis were performed.

Postoperative follow-up included medical examination, blood tests with serum CA 19–9 level, and CT examination every 3 months for the first 3 years, then every 6 months for the next 2 years, and then annually thereafter.

### SMV measurement

**Fig. 2:**
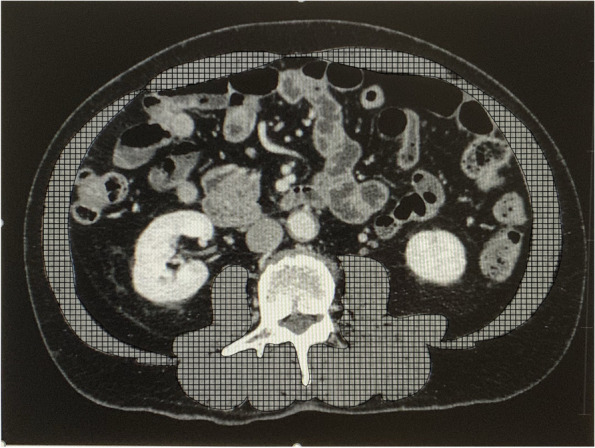
Evaluation of skeletal muscle mass volume. Axial computed tomography image of the abdomen at the third lumbar vertebra. The skeletal muscle area is highlighted in hatching (Hounsfield unit threshold from − 29 to + 150)

As shown in Fig. 2, SMV was measured at the third lumbar vertebra using a Hounsfield unit range of − 29 to 150 on CT axial images. A single investigator (S.Y.), who was blinded to the patients’ clinical histories and outcomes, assessed the images and manually traced the skeletal muscle area. The skeletal muscle index (SMI, cm^2^·m^− 2^) for each patient was calculated as follows: SMI = (skeletal muscle area) / (height).^2^ Sarcopenia was defined as SMI < 43 cm^2^·m^− 2^ in men with a body mass index < 25 kg·m^− 2^, SMI < 53 cm^2^·m^− 2^ in men with a body mass index ≥25 kg·m^− 2^, or SMI < 41 cm^2^·m^− 2^ in women [19]. All studied patients underwent CT examination within 30 days prior to surgery (preoperative period), 3–6 months after surgery (early postoperative period), and 12 months after surgery (late postoperative period). The percent change of the SMI in each patient between the preoperative period and the early or late postoperative period was calculated. CT images were analyzed by Volume Analyzer SYNAPSE VINCENT (Fujifilm Medical, Tokyo, Japan).

### Statistical analysis

Categorical variables are presented as n (%) and were analyzed using Fisher’s exact test. Continuous variables are presented as median [range] and were analyzed using the Mann-Whitney U-test. Cutoffs were determined as the upper limit of the normal range for laboratory data and as the median for other continuous variables. Correlations between the variables were evaluated using Spearman’s rank correlation coefficient. Overall survival (OS) was defined as the time from surgery to death or the date at last follow-up. Recurrence-free survival (RFS) was calculated as the time from surgery to recurrence, death, or the date at last follow-up. Recurrence was defined as the detection of a tumor on imaging or by pathological confirmation.

The study observation period was until December 2019. OS and RFS were determined by Kaplan-Meier survival analysis and the log-rank test. Clinicopathological findings and SMI were evaluated for the associations with a shorter OS or RFS by calculating hazard ratios (HRs) and 95% confidence intervals (95% CIs) using Cox regression analysis. Variables that were shown to be significant in univariable analysis were included in multivariable analysis, excluding the confounder with a lower hazard ratio if a correlation was shown between variables with Spearman’s rank analysis. The significant correlation coefficient was set at r _s_ > 0.3. Variables in the early and late postoperative periods were evaluated separately because risk analysis for a shorter OS or RFS should be performed using the variables of each early and late postoperative period. The associations of decreased SMI and elevated serum CA 19–9 level in the early and late postoperative period with recurrence and poor survival was compared. All *P*-values were based on two-sided statistical tests, and the significance level was set at *P* <  0.05. All statistical analyses were performed using JMP version 13 (SAS Institute, Cary, NC, USA).

## Results

### Clinicopathological characteristics

Clinicopathological characteristics are shown in Table 1. Median patient age was 70 [39–81] years, and 38 patients (68%) were male. Thirty-four patients (61%) underwent hepatectomy of more than 4 segments. Ten patients (18%) underwent concomitant portal vein resection and reconstruction and 1 patient (2%) underwent concomitant pancreaticoduodenectomy. There were 16 patients (29%) with a pathological T3 or T4 disease and 23 patients (41%) with lymph node metastasis. Twenty-four patients (43%) had R1 resection, and there were no patients with R2 resection. During the median follow-up of 57.9 months (95% CI, 47.9–84.3 months), 35 patients (63%) developed recurrence and 29 patients (50%) died. One patient developed recurrence within 6 months after surgery and 3 patients did within 12 months after surgery.

**Table 1:** Clinicopathological characteristics

Preoperative variables	
Age, years	70 [39–81]
Sex, male	38 (68%)
BMI, kg·m^− 2^	21.4 [15.0–30.5]
ASA score ≥ 2	44 (79%)
Charlson comorbidity index ≥ 2	5 (9%)
Preoperative biliary drainage	38 (68%)
Portal vein embolization	31 (55%)
Serum total bilirubin, mg/dL	0.9 [0.3–4.0]
Serum albumin, g/dL	3.7 [2.7–5.0]
Serum CA 19–9, U/mL	58.9 [0.2–6121.0]
Intraoperative variables	
Hepatectomy of more than 4 segments	34 (61%)
Operation time, min	500 [317–760]
Blood loss, mL	1423 [270–6756]
Blood transfusion	26 (46%)
Postoperative course	
Major complications (Clavien grade ≥ IIIa)	22 (39%)
Surgical site infection	26 (46%)
Bile leakage (ISGLS grade B/C)	9 (16%)
Liver failure (ISGLS grade B/C)	13 (23%)
Length of hospitalization, days	20 [10–70]
Pathological data	
Tumor size, mm	26 [9–65]
T3/4 (UICC 8th)	16 (29%)
Lymph node metastasis	23 (41%)
R1 resection	24 (43%)

*ASA* American Society of Anesthesiologists classification, *BMI* body mass index, *CA 19–9* carbohydrate antigen 19–9, *EBDR* extrahepatic bile duct resection and reconstruction, *ISGLS* International Study Group of Liver Surgery, *PD* pancreaticoduodenectomy, *UICC* Union for International Cancer Control

### SMI

In the preoperative period, median SMI was 44.4 [30.9–57.2] cm^2^·m^− 2^ and 26 patients (46%) were categorized as having sarcopenia. In the early postoperative period (median of 137 days), median SMI was 45.1 [32.0–57.3] cm^2^·m^− 2^ and 23 patients (41%) had sarcopenia. In comparison between preoperative and early postoperative periods, the median percent change of SMI was − 0.16% [− 10.4 to 14.5], and SMI decreased in 29 patients (52%). In the late postoperative period (median of 372 days), median SMI was 43.7 [31.6–57.6] cm^2^·m^− 2^ and 28 patients (50%) had sarcopenia. In comparison between preoperative and late postoperative periods, the median percent change of SMI was − 0.59% [− 17.4 to 21.8], and SMI decreased in 29 patients (52%).

### Risk analysis for a shorter OS

As shown in Table 2, clinicopathological variables, including decreased SMI and elevated serum CA 19–9 level in the preoperative and early or late postoperative period, were evaluated for the associations with a shorter OS. In the early postoperative period (multivariable model 1), only decreased SMI (HR, 2.39; 95% CI, 1.00–6.18; *P* = 0.049) was independently associated with a shorter OS. In the late postoperative period (multivariable model 2), decreased SMI (HR, 2.28; 95% CI, 1.02–5.50; *P* = 0.045) and elevated serum CA 19–9 level (HR, 6.54; 95% CI, 2.59–17.57; *P* <  0.001) were independently associated with a shorter OS. Of note, sarcopenia in the late postoperative period was excluded from multivariable model 2 because it was correlated with decreased SMI in the late postoperative period (r _s_ = 0.350; *P* = 0.007).

**Table 2:** Analysis of risk factors for shorter OS

	Univariable analysis		Multivariable analysis			
			Model 1 Early postoperative period		Model 2 Late postoperative period	
	HR [95% CI]	*P*	HR [95% CI]	*P*	HR [95% CI]	*P*
Preoperative variables						
Age > 70 years	1.41 [0.66–2.99]	0.368				
Sex, male	0.95 [0.44–2.22]	0.907				
BMI > 25 kg·m^−2^	1.61 [0.59–3.78]	0.330				
ASA score ≥ 2	0.96 [0.43–2.45]	0.933				
Charlson comorbidity index ≥ 2	1.82 [0.29–6.27]	0.458				
Preoperative biliary drainage	1.09 [0.51–2.53]	0.832				
Portal vein embolization	1.04 [0.49–2.24]	0.918				
Serum total bilirubin > 1.2 mg/dL	0.82 [0.34–1.81]	0.638				
Serum albumin < 3.8 g/dL	0.84 [0.40–1.79]	0.655				
Serum CA 19–9 > 37 U/mL	1.45 [0.68–3.27]	0.342				
Sarcopenia	1.10 [0.52–2.33]	0.798				
Intraoperative variables						
Hepatectomy of more than 4 segments	1.07 [0.48–2.49]	0.868				
Operation time > 500 min	2.83 [1.27–6.78]	0.010	1.74 [0.74–4.40]	0.210	1.61 [0.69–4.02]	0.275
Blood loss > 1500 mL	1.20 [0.57–2.57]	0.623				
Blood transfusion	0.95 [0.42–2.10]	0.891				
Postoperative course						
Major complications (Clavien grade ≥ IIIa)	1.39 [0.65–2.96]	0.389				
Surgical site infection	1.45 [0.68–3.10]	0.333				
Bile leakage (ISGLS grade B/C)	0.78 [0.23–2.03]	0.635				
Liver failure (ISGLS grade B/C)	1.16 [0.46–2.62]	0.734				
Length of hospitalization ≥ 20 days	1.70 [0.79–3.76]	0.172				
Pathological data						
Tumor size > 25 mm	1.17 [0.55–2.49]	0.687				
T3/4 (UICC 8th)	2.40 [1.04–6.41]	0.040	1.91 [0.82–4.23]	0.128	1.79 [0.75–5.50]	0.185
Lymph node metastasis	2.86 [1.33–6.41]	0.008	2.08 [0.94–4.77]	0.071	1.44 [0.29–3.43]	0.404
R1 resection	1.90 [0.90–4.06]	0.093				
Early postoperative period						
Serum CA 19–9 > 37 U/mL	1.97 [0.72–4.63]	0.171				
Sarcopenia	1.70 [0.81–3.62]	0.160				
Decreased SMI	3.44 [1.55–8.41]	0.002	2.39 [1.00–6.18]	0.049		
Late postoperative period						
Serum CA 19–9 > 37 U/mL	9.48 [3.94–24.04]	< 0.001			6.54 [2.59–17.57]	< 0.001
Sarcopenia	2.28 [1.08–5.04]	0.031				
Decreased SMI	3.15 [1.45–7.40]	0.004			2.28 [1.02–5.50]	0.045

*95% CI* 95% confidence interval, *ASA* American Society of Anesthesiologists classification, *BMI* body mass index, *CA 19–9* carbohydrate antigen 19–9, *HR* hazard ratio, *ISGLS* International Study Group of Liver Surgery; OS, overall survival, *SMI* skeletal muscle index, *UICC* Union for International Cancer Control

### Risk analysis for a shorter RFS

As shown in Table 3, clinicopathological variables were evaluated for the associations with a shorter RFS. In the early postoperative period (multivariable model 1), lymph node metastasis (HR, 2.01; 95% CI, 1.01–4.04; *P* = 0.047) and decreased SMI (HR, 2.14; 95% CI, 1.04–4.57; *P* = 0.039) were independently associated with a shorter RFS. In the late postoperative period (multivariable model 2), only elevated serum CA 19–9 level (HR, 4.55; 95% CI, 2.10–9.96; *P* <  0.001) was independently associated with a shorter RFS.

**Table 3:** Analysis of risk factors for shorter RFS

	Univariable analysis		Multivariable analysis			
			Model 1 Early postoperative period		Model 2 Late postoperative period	
	HR [95% CI]	*P*	HR [95% CI]	*P*	HR [95% CI]	*P*
Preoperative variables						
Age > 70 years	1.06 [0.56–2.02]	0.861				
Sex, male	1.04 [0.54–2.15]	0.904				
BMI > 25 kg·m^−2^	1.30 [0.55–2.72]	0.524				
ASA score ≥ 2	0.87 [0.43–1.96]	0.723				
Charlson comorbidity index ≥ 2	1.27 [0.30–3.55]	0.707				
Preoperative biliary drainage	1.18 [0.61–2.42]	0.638				
Portal vein embolization	1.27 [0.66–2.51]	0.475				
Serum total bilirubin > 1.2 mg/dL	0.73 [0.34–1.47]	0.390				
Serum albumin < 3.8 g/dL	1.06 [0.56–2.06]	0.859				
Serum CA 19–9 > 37 U/mL	1.21 [0.64–2.38]	0.558				
Sarcopenia	0.96 [0.43–1.71]	0.748				
Intraoperative variables						
Hepatectomy of more than 4 segments	1.47 [0.75–3.08]	0.268				
Operation time > 500 min	2.17 [1.13–4.28]	0.020	1.53 [0.76–3.13]	0.235	1.67 [0.85–3.37]	0.136
Blood loss > 1500 mL	1.72 [0.90–3.33]	0.098				
Blood transfusion	1.48 [0.78–2.90]	0.233				
Postoperative course						
Major complications (Clavien grade ≥ IIIa)	1.53 [0.80–2.90]	0.196				
Surgical site infection	1.44 [0.76–2.76]	0.261				
Bile leakage (ISGLS grade B/C)	0.94 [0.38–2.01]	0.876				
Liver failure (ISGLS grade B/C)	1.52 [0.72–3.00]	0.257				
Length of hospitalization ≥ 20 days	1.60 [0.84–3.16]	0.153				
Pathological data						
Tumor size > 25 mm	1.13 [0.59–2.15]	0.714				
T3/4 (UICC 8th)	2.02 [0.99–3.91]	0.053				
Lymph node metastasis	2.68 [1.40–5.21]	0.003	2.01 [1.01–4.04]	0.047	1.62 [0.79–3.34]	0.186
R1 resection	1.86 [0.98–3.55]	0.059				
Early postoperative period						
Serum CA 19–9 > 37 U/mL	1.75 [0.71–3.76]	0.209				
Sarcopenia	1.40 [0.73–2.66]	0.309				
Decreased SMI	2.91 [1.50–5.90]	0.001	2.14 [1.04–4.57]	0.039		
Late postoperative period						
Serum CA 19–9 > 37 U/mL	6.42 [3.10–13.24]	< 0.001			4.55 [2.10–9.96]	< 0.001
Sarcopenia	1.81 [0.95–3.50]	0.070				
Decreased SMI	2.27 [1.18–4.52]	0.014			1.25 [0.60–2.66]	0.549

*95% CI* 95% confidence interval, *ASA* American Society of Anesthesiologists classification, *BMI* body mass index, *CA 19–9* carbohydrate antigen 19–9, *HR* hazard ratio, *ISGLS* International Study Group of Liver Surgery, *RFS* recurrence-free survival, *SMI* skeletal muscle index, *UICC* Union for International Cancer Control

### Associations of decreased SMI and serum CA 19–9 level in the early/late postoperative periods with OS and RFS

**Fig. 3:**
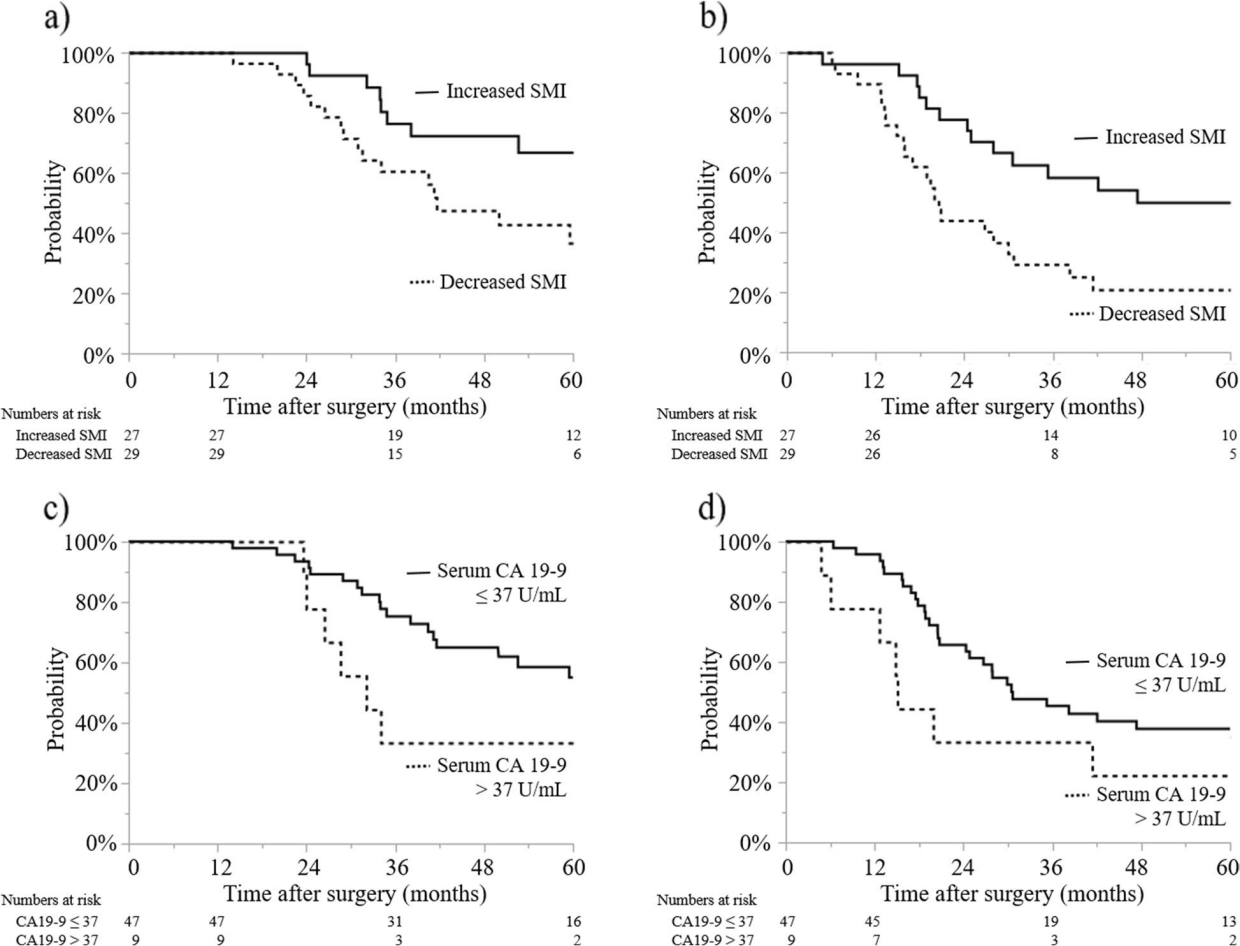
Overall and recurrence-free survival with respect to changes in skeletal muscle index (SMI) or serum carbohydrate antigen 19–9 (CA 19–9) levels in the early postoperative period. **a** Overall survival of patients in whom SMI decreased or increased postoperatively (42 months [95% confidence interval (95% CI), 31–64] vs. not reached (NR) [95% CI, 53-NR], *P* = 0.002). **b** Recurrence-free survival of patients in whom SMI decreased or increased postoperatively (20 months [95% CI, 16–31] vs. NR [95% CI, 55-NR], *P* = 0.001). **c** Overall survival of patients with serum CA 19–9 > 37 or ≤ 37 U/mL (32 months [95% CI, 24-NR] vs. 64 months [95% CI, 42-NR], *P* = 0.136). **d** Recurrence-free survival of patients with serum CA 19–9 > 37 or ≤ 37 U/mL (15 months [95% CI, 5-NR] vs. 31 months [95% CI, 24–60], *P* = 0.176)

As shown in Fig. 3, the associations of OS and RFS with SMI and serum CA 19–9 level in the early postoperative period were evaluated. Twenty-nine patients (52%) exhibited decreased SMI and 9 patients (16%) had elevated serum CA 19–9 levels. Among all patients, the median serum CA 19–9 level was 16.2 U/mL [0.1–246.3 U/mL]. Compared to patients in whom SMI increased, those in whom SMI decreased had a shorter OS (42 months vs. not reached (NR), *P* = 0.002; Fig. 3a) and a shorter RFS (20 months vs. NR, *P* = 0.001; Fig. 3b). Neither OS (32 vs. 64 months, *P* = 0.136; Fig. 3c) nor RFS (15 vs. 31 months, *P* = 0.176; Fig. 3d) differed significantly between patients with and without elevated serum CA 19–9 levels.

**Fig. 4:**
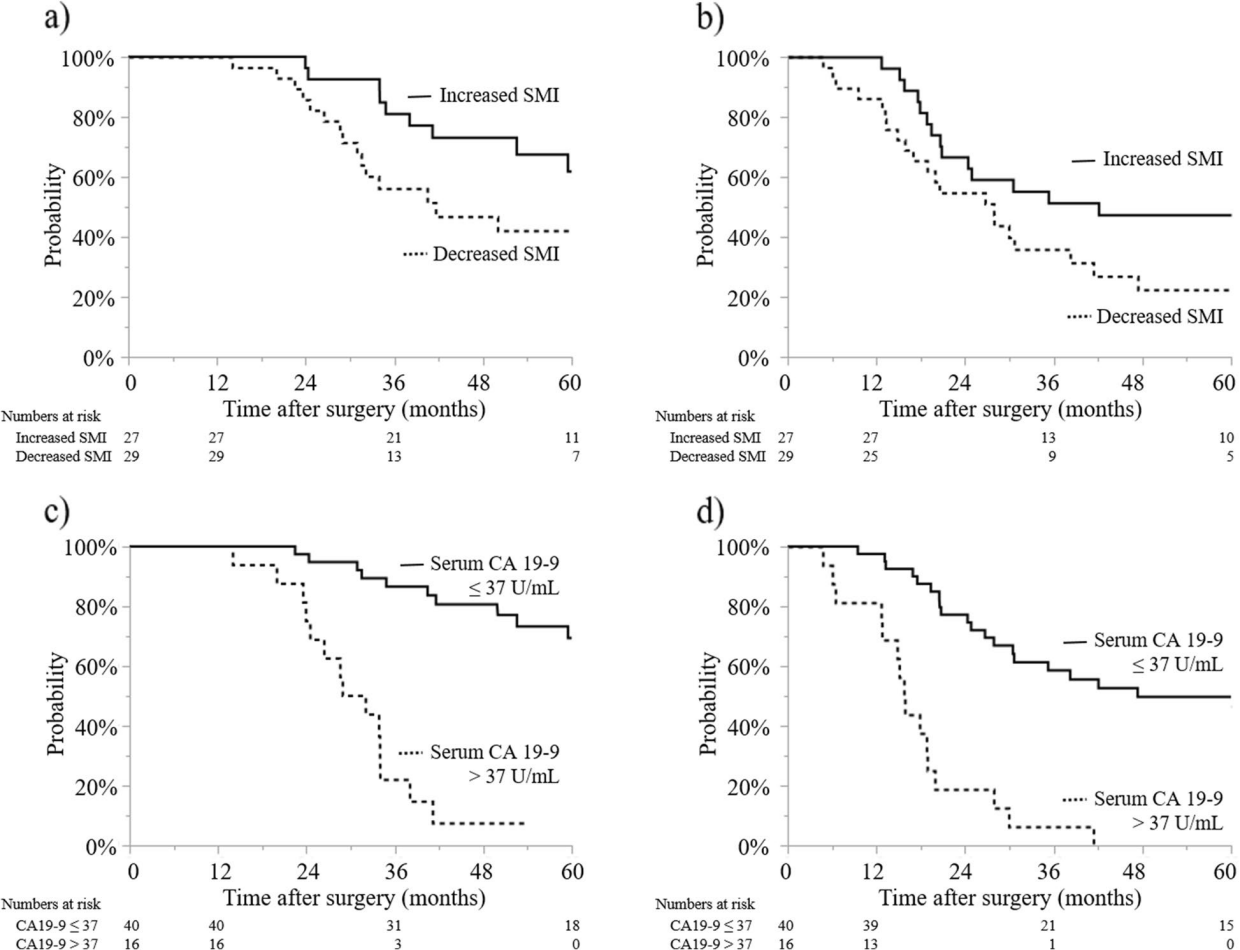
Overall and recurrence-free survival with respect to changes in skeletal muscle index (SMI) or serum carbohydrate antigen 19–9 (CA 19–9) levels in the late postoperative period. **a** Overall survival of patients in whom SMI decreased or increased postoperatively (42 months [95% confidence interval (95% CI), 31–64] vs. not reached (NR) [95% CI, 52-NR], *P* = 0.003). **b** Recurrence-free survival of patients in whom SMI decreased or increased postoperatively (28 months [95% CI, 16–38] vs. 42 months [95% CI, 21-NR], *P* = 0.013). **c** Overall survival of patients with serum CA 19–9 > 37 or ≤ 37 U/mL (31 months [95% CI, 24–34] vs. 87 months [95% CI, 63-NR], *P* <  0.001). **d** Recurrence-free survival of patients with serum CA 19–9 > 37 or ≤ 37 U/mL (16 months [95% CI, 13–19] vs. 47 months [95% CI, 28-NR], *P* <  0.001). *P* = 0.003)

As shown in Fig. 4, in the late postoperative period, 29 patients (52%) exhibited decreased SMI and 16 patients (29%) had elevated serum CA 19–9 levels. Among all patients, the median serum CA 19–9 level was 16.8 U/mL [0.4–1654.0 U/mL]. Compared to patients in whom SMI increased, those in whom SMI decreased had a shorter OS (42 months vs. NR, *P* = 0.003; Fig. 4a) and a shorter RFS (28 vs. 42 months, *P* = 0.013; Fig. 4b). Compared to patients without elevated serum CA 19–9 levels, those with elevated serum CA 19–9 levels had a shorter OS (31 vs. 87 months, *P* <  0.001; Fig. 4c) and a shorter RFS (16 vs. 47 months, *P* <  0.001; Fig. 4d).

## Discussion

In this study, 46% of patients who underwent major hepatectomy with EBDR for perihilar cholangiocarcinoma had sarcopenia preoperatively; among the studied patients SMI decreased in 52% in the early postoperative period and also in 52% in the late postoperative period. Preoperative sarcopenia was not associated with a shorter OS or RFS. However, decreased SMI in the early postoperative period was independently associated with both shorter OS and RFS. Moreover, compared to elevated serum CA 19–9 level, decreased SMI in the early postoperative period was associated more strongly with recurrence and poor survival. On the other hand, decreased SMI in the late postoperative period was independently associated with a shorter OS but not with a shorter RFS.

Tran et al. found that elevated preoperative serum CA 19–9 level, lymph node metastasis, and R1 resection were associated with poorer 5-year survival among 194 patients who underwent perihilar cholangiocarcinoma resection [3]. Zhang et al. systematically reviewed 38 cohort studies and found that lymph node metastasis, positive resection margin, intraoperative transfusion, pathological stage T3/4, and moderately or poorly differentiated adenocarcinoma were associated with poor prognosis in patients with perihilar cholangiocarcinoma [9]. It should be noted that all variables evaluated in those studies were determined preoperatively or from pathological findings, whereas the present study evaluated variables determined pre-, intra-, and post-operatively.

One study evaluated the associations of the perioperative percent change in SMV with postoperative outcomes in patients who underwent major hepatectomy with EBDR for perihilar cholangiocarcinoma at a high-volume center in Japan [19]. The authors measured the total psoas muscle area at the third lumbar vertebra on CT both preoperatively and 1 week postoperatively and showed that total psoas muscle area was significantly lower postoperatively than preoperatively, with a median percent change of − 2.2%. Furthermore, in that study, patients in whom total psoas muscle area decreased postoperatively had a significantly higher incidence of major postoperative complications and surgery-related mortality than those in whom total psoas muscle area did not decrease postoperatively. The authors described that skeletal muscle degradation 1 week postoperatively was due to the release of inflammatory cytokines, such as plasma interleukin (IL)-6 or tumor necrosis factor-α (TNF-α), triggered by surgery or postoperative complications.

Several studies evaluated the mechanism for the poor prognosis of patients in whom SMV decreased in the clinical course of malignant disease [20, 21]. Lutz et al. showed that IL-15, a myokine released by muscle, affects the development and survival of natural killer cells and negatively regulates adipose tissue to release inflammatory cytokines such as IL-6 and TNF-α, which inhibit the survival and activities of natural killer cells [21]. Accordingly, they described that a decrease in SMV consequently reduces the number and functions of natural killer cells and attenuates antitumor responses, resulting in poor prognosis for patients with malignant diseases.

Serum CA 19–9 is usually measured during follow-up after surgery for perihilar cholangiocarcinoma [10]; its elevation is reported to be a risk factor for early recurrence and poor survival [22]. Moreover, other previous study reported that serum CA19–9 was obviously superior to serum CEA in the diagnosis of cholangiocarcinoma and often considered the standard marker for the postoperative follow-up of cholangiocarcinoma, with which other markers were compared [23]. In the present study, elevated serum CA 19–9 level in the late postoperative period, instead of the early postoperative period, was significantly associated with a shorter OS and RFS. In contrast, decreased SMI in the early postoperative period was significantly associated with a shorter OS and RFS.

These results collectively suggest that decreased SMI is a more useful predictor for early recurrence and poor survival than elevated serum CA 19–9 level. Thus, measurement of SMI was considered to be useful for monitoring patients after surgery, and a decrease in SMI might be an early sign of tumor progression that precedes the elevation of tumor markers and appearance of tumor on CT. Accordingly, detecting recurrence before lesions on radiological examination enables clinicians to plan closer follow-up or additional examinations, or start secondary treatment. Moreover, the initiation of systemic chemotherapy at an earlier disease stage might improve survival.

One limitation of this study was its retrospective design, which introduces a risk of selection bias mainly due to the exclusion of patients who were not surgical candidates. Second, this study included a small sample size from a single institution, as surgery for patients with perihilar cholangiocarcinoma is generally rare owing to its incidence, difficulty in diagnosis, and invasiveness and risks in surgery, even in a high-volume center. Further, multi-center investigations to analyze more cases would be recommended. Third, secondary treatments after recurrence were not considered in survival analysis. However, patients who were receiving pre- or postoperative adjuvant therapy as part of a clinical trial were excluded because the presence of adjuvant therapy might affect survival outcomes, regardless of the change of SMI. Clinical variables that were associated especially with early recurrence in condition with no other anti-cancer therapy than surgery were sought in this study.

## Conclusions

This study showed that a decreased SMI in the early postoperative period was independently associated with a shorter OS and RFS in patients who underwent major hepatectomy with EBDR for perihilar cholangiocarcinoma. During the early postoperative period in particular, decreased SMI appeared to be a better predictor for recurrence and poor survival than elevated serum CA 19–9 level. Hence, evaluating SMI on serial CTs was considered to be useful for monitoring patients after surgery.

## Data Availability

We promise to that the materials described in the manuscript, including all relevant raw data, will be freely available to any scientist wishing to use them for non-commercial purposes, without breaching participant confidentiality. The datasets used and/or analyzed during the current study are available from the corresponding author on reasonable request.

## Abbreviations

EBDR: Extrahepatic bile duct resection and reconstruction
SMV: Skeletal muscle mass volume
UICC: Union for International Cancer Control
CA19–9: Carbohydrate antigen 19–9
CT: Computed tomography
SMI: Skeletal muscle index
OS: Overall survival
RFS: Recurrence-free survival
HR: Hazard ratio
95% CI: 95% confidence interval
NR: Not reached
IL: Interleukin
TNF-α: Tumor necrosis factor-α
BMI: Body mass index
ISGLS: International Study Group of Liver Surgery
